# Penicillin Allergy Assessment and Skin Testing in the Outpatient Setting

**DOI:** 10.3390/pharmacy7030136

**Published:** 2019-09-19

**Authors:** Wesley D. Kufel, Julie Ann Justo, P. Brandon Bookstaver, Lisa M. Avery

**Affiliations:** 1 Department of Pharmacy Practice, Binghamton University School of Pharmacy and Pharmaceutical Sciences, Binghamton, NY 13902, USA; wkufel@binghamton.edu; 2 Department of Medicine, State University of New York Upstate Medical University, Syracuse, NY 13210, USA; 3 Department of Pharmacy, State University of New York Upstate University Hospital, Syracuse, NY 13210, USA; 4 Department of Clinical Pharmacy and Outcomes Sciences, University of South Carolina College of Pharmacy, Columbia, SC 29208, USA; justoj@cop.sc.edu (J.A.J.); bookstap@email.sc.edu (P.B.B.); 5 Department of Pharmacy, Prisma Health Richland Hospital, Columbia, SC 29203, USA; 6 Department of Pharmacy Practice, Wegmans School of Pharmacy, St. John Fisher College, Rochester, NY 14618, USA; 7 Department of Pharmacy, St. Josephs Health, Syracuse, NY 13203, USA

**Keywords:** penicillin, allergy, skin testing, outpatient, pharmacist, antimicrobial stewardship

## Abstract

Penicillin allergies are among of the most commonly reported allergies, yet only 10% of these patients are truly allergic. This leads to potential inadvertent negative consequences for patients and makes treatment decisions challenging for clinicians. Thus, allergy assessment and penicillin skin testing (PST) are important management strategies to reconcile and clarify labeled penicillin allergies. While PST is more common in the inpatient setting where the results will immediately impact antibiotic management, this process is becoming of increasing importance in the outpatient setting. PST in the outpatient setting allows clinicians to proactively de-label and educate patients accordingly so beta-lactam antibiotics may be appropriately prescribed when necessary for future infections. While allergists have primarily been responsible for PST in the outpatient setting, there is an increasing role for pharmacist involvement in the process. This review highlights the importance of penicillin allergy assessments, considerations for PST in the outpatient setting, education and advocacy for patients and clinicians, and the pharmacist’s role in outpatient PST.

## 1. Introduction

Penicillin allergy is the most commonly reported antibiotic allergy, with approximately 10% of the general population being labeled as penicillin allergic [1]. However, nearly 90% of patients with reported penicillin allergies are not type I IgE-mediated reactions [1,2]. Documentation of inaccurate penicillin allergies is associated with unintended negative consequences, especially for common infections in the outpatient setting where treatment with beta-lactam antibiotics is preferred in both pediatric and adult patients [3]. These include increased use of alternative or broader spectrum antibiotics with the potential for additional adverse effects including *Clostridioides difficile*-associated diarrhea (CDAD), increased medical costs, worse clinical outcomes, or increased development of bacterial resistance [4,5,6,7]. While the majority of penicillin allergy outcome data is from the inpatient setting, patients with a penicillin allergy in the outpatient setting are also more likely to receive additional antibiotics, have higher antibiotic costs, and utilize more healthcare resources [8,9].

One of the primary issues is that penicillin allergies are often misdiagnosed or mislabeled as true allergies, and when present, diminish over time in most patients [10]. Clinicians and patients are frequently unaware that penicillin allergies are misdiagnosed and that patients may be able to still tolerate penicillin or other beta-lactam antibiotics. This is an opportunity for pharmacist involvement in the outpatient setting to perform comprehensive penicillin allergy assessments, increase referrals for penicillin skin testing (PST), perform PST, educate clinicians and patients, and ensure appropriate allergy documentation [11,12]. PST is a reasonable, safe procedure that is already being performed in outpatient allergy clinics, but has a potential to reach even more patients with pharmacist involvement. This public health initiative would allow patients to be de-labeled and proactively made aware of their penicillin allergy history so that beta-lactam antibiotics can be appropriately prescribed when needed for a future infection in either an outpatient or inpatient setting. Therefore, the focus of this article is to review and highlight penicillin allergy assessment and PST opportunities in the outpatient setting. This review includes references identified via PubMed using the search terms “skin tests”, “penicillin”, and “outpatient”, but is not a comprehensive review of all available literature. The focus is on areas where pharmacists can improve the care of a patient labeled with a penicillin allergy. A detailed review of penicillin allergy assessment and PST in the inpatient setting, including the PST process, is described elsewhere [13].

## 2. Penicillin Allergy Interview Assessment

Appropriate assessment of reported penicillin allergies should begin with a thorough interview, including the following components: characterization of the reaction, likelihood of causality, IgE- vs. non-IgE-mediated reaction, timeframe of the reaction, receipt of other antibiotics, and history of skin testing and other drug allergy testing [1,10,14]. These questions are essential to risk stratify the likelihood of a true penicillin allergy and options for reconciliation. This interview is an excellent opportunity to provide education to patients on the potential negative consequences of reported penicillin allergies and the statistics on the number of patients who report penicillin allergies compared to those that are truly allergic [1,2,3]. Many patients may be labeled with a penicillin allergy, but they may have received a similar antibiotic in the past such as amoxicillin or another oral penicillin-type antibiotic. It is important to recognize the specific antibiotic that the patient received since there are different cross-reactivity potentials across the beta-lactam antibiotics based on similarities in their R-1 side chain of their chemical structure [15,16]. A discussion on R-1 side chains and cross-reactivity among beta-lactam antibiotics is outside the scope of this article; however, Chaudhry and colleagues provide a thorough review on this topic elsewhere [17]. A detailed description of the reaction is also necessary since some reactions may be intolerances rather than true allergies. Additionally, this helps to determine the likelihood of causality to penicillin and stratify if patients experienced a type I IgE-mediated reaction or if it was a less severe reaction. Patients may get confused with allergic reaction terminology and may not be able to differentiate between a less severe reaction such as a maculopapular rash versus a potentially severe reaction such as hives. Patients may also have difficulty in recalling the specific details of the reaction, particularly if the reaction was several years ago or was deemed low severity. Thus, pharmacists should consider asking patients to describe the reaction in an open-ended fashion, then follow-up with closed-ended questions which elucidate the differences between these reactions [14]. This should result in the most accurate description of the reaction.

Patients who had a previous reaction may not have a subsequent reaction due to waning immunity over time, especially among patients where the reaction occurred decades ago [1]. Approximately 80% of patients lose sensitivity of their initial, true allergic reaction after 10 years [10]. Thus, it is important for pharmacists and patients to identify the timeframe of the penicillin reaction. Many patients are also frequently unaware they may have received and tolerated antibiotics similar to penicillin. However, patients may not able to recall the specific beta-lactam antibiotic they received. Therefore, pharmacists should consider listing some common oral beta-lactam antibiotics using both the generic and brand name, where appropriate, in an effort to increase the likelihood of recollection. Pharmacists and patients may also review pharmacy records to assess antibiotic prescription history. Despite these efforts, patients may still not be able to accurately answer all of these questions, which makes appropriate assessment for clinicians challenging, especially when many of the answers to the questions are unknown. In patients deemed higher risk for a true allergic reaction or with an unknown allergy history, PST is a helpful management option in the outpatient setting [18,19,20,21]. 

## 3. Clinicians Involved in PST

PST in the outpatient setting has historically been performed in allergist-run specialty clinics [22,23]. Pharmacists currently have a limited role, especially in outpatient PST [18]. A survey of Boards of Pharmacy in 2017 revealed that only 18 states allowed pharmacists to perform PST based on their state’s scope of practice, with an additional 8 states potentially allowing permission based on certain requirements and regulations [18]. For example, New York State’s current legislation allows for the administration of intramuscular injections, but does not allow pharmacists to administer intradermal injections. As such, PST is a great opportunity for professional pharmacy organizations at the state and national level to advocate for pharmacists to perform PST, especially with The Joint Commission’s requirement for expansion of antimicrobial stewardship in ambulatory care settings in 2020 [24]. In states where pharmacists are not allowed to perform PST, ambulatory care and infectious diseases pharmacists can still play an essential role in collaborative patient referral opportunities for outpatient PST, consideration of direct oral challenges for select patients, and the provision of patient and provider education surrounding penicillin allergy.

## 4. Referrals for PST

A proactive, elective referral to an allergist or clinic performing PST in the outpatient setting helps avoid potential complications associated with a penicillin allergy history during hospitalization. In 2017, a position statement from the Penicillin Allergy in Antibiotic Resistance Workgroup of the American Academy of Allergy, Asthma, & Immunology recommended routine PST in patients with a self-reported penicillin allergy [25]. Despite this recommendation, routine PST is not common practice. A study of an urban, adult outpatient clinic referred only 6% (78/1348) of penicillin allergic patients to an allergy specialist [26]. Barriers to elective referrals included lack of knowledge regarding PST availability and limited clinics that were accessible to perform PST in various areas.

There are no clinical studies currently available that focus on successful penicillin allergy referral programs by pharmacists. The majority of studies focus on preoperative allergy assessment in the cardiac and orthopedic populations [27,28]. A systematic review and meta-analysis identified four studies that observed a significantly lower rate of non-beta-lactam antibiotic usage when preoperative antibiotic allergy testing protocols were employed compared to standard of care (odds ratio of 3.64 [95% confidence interval, 2.67–4.98]; *p* < 0.0001) [29]. A recent study by Wyles and colleagues reviewed preoperative antibiotics for total knee and hip arthroplasties and the impact of antibiotic allergy testing on cefazolin use and subsequent infection rates [30]. A total of 97% (2493/2576) of PST tested patients were cleared to receive cefazolin as their antibiotic prophylaxis regimen. The increased use of cefazolin resulted in a 32% lower rate of prosthetic joint infections. Based on these data, the authors recommended perioperative testing and clearance in all orthopedic patients with either a penicillin or a cephalosporin allergy.

In the non-surgical population, routine penicillin allergy referral to a PST provider is limited. The Miami Veterans Affairs Health Care System proactively referred outpatients with a self-reported penicillin allergy to their allergy clinic for PST testing. Of 41 veterans who underwent testing, 93% were PST negative, which resulted in a 39% reduction in broad-spectrum antibiotic use over a 3-year follow-up period [31]. Other potential target populations for PST referrals include patients in long-term care facilities (LTCF), urgent care clinics, pediatric clinics, and emergency departments. Many patients residing in LTCF with labeled penicillin allergies are likely to be prescribed alternative antibiotics to beta-lactams for infections, such as fluoroquinolones. This alternative exposure is of particular concern given this patient population may be even more susceptible to potential adverse effects from fluroquinolones [32]. As such, this is an area for consultant pharmacists and LTCF clinicians to proactively assess penicillin allergies and perform PST, where appropriate, to potentially prevent CDAD and other adverse effects secondary to alternative antibiotic use.

Urgent care clinics are often recognized as a convenient resource for many patients to seek healthcare services for acute care issues including infections. However, many patients with penicillin allergies who seek urgent care clinics for infectious-related problems may not have the same referral opportunity and provider relationship since many of these patients may not have an established primary care provider [33]. Pediatric patients also seek acute care visits for common infections, such as acute otitis media, where beta-lactams are considered standard of care; however, penicillin allergies can preclude the optimal therapeutic recommendations in this patient population [34]. As such, PST in these outpatient settings is important to consider despite limited available data. In addition to provider referrals, there may also be a role for healthcare plan referral to capitalize on lower utilization of healthcare resources associated with a negative PST. Macy and colleagues identified an annual savings per patient of $1915.40 in healthcare expenditures in a patient population with higher baseline healthcare utilization [35].

## 5. Time, Cost, and Reimbursement Considerations for PST

### 5.1. Clinician Time

The time dedicated by pharmacists to perform PST is dependent on the testing performed, personnel involved in the preparation of the products, and involvement of ancillary staff [18]. To calculate the pharmacist time involved with PST, there needs to be an estimate of the time required to perform an accurate and comprehensive allergy history interview, prepare the PST contents, perform a prick test with a 15-min wait time for results, and perform intradermal testing with a 15-min wait time for results. If all test results are negative, clinicians may choose to perform a direct, oral challenge with amoxicillin. This requires an additional 60 min or more for patient monitoring. After the completion of the PST, patient education time, documentation time, and dedicated time to inform other providers of results should also be included. This time would also be prolonged if the patient developed an adverse reaction to the PST procedure. However, the likelihood of a systemic reaction occurring from PST is rare and has been reported as 0.16% among nearly 20,000 patients exposed to both major and minor determinants [36].

The time required to perform a detailed allergy history is highly variable, with one allergy group calculating an average of 13.8 min [37]. Drug mixing time typically requires less than 5 min, but could be longer if batching is performed. A recent survey of North American providers estimated a median time of 20 min to perform the prick and intradermal test respectively [38]. A direct, oral challenge can be performed with a one- or two-step method. A one-step method involves administration of a therapeutic dose of amoxicillin and monitoring patients for 60 min or more. A two-step method involves giving the patient a fraction (10%) of a therapeutic dose with a 30-min observation period followed by administration of the full therapeutic dose (90%) with 60-min post-observation time. Overall, the total time required is variable based on the patient history, testing procedure performed, and clinic workflow, but can range anywhere from 45–120 min [37].

### 5.2. Supply Cost

The cost of PST supplies is approximately $140 to $160 per patient (Table 1). The following rescue medications are not included in this per patient cost estimate: epinephrine auto-injector device, diphenhydramine oral, prednisone/methylprednisolone oral, and albuterol inhaler. Labor and supply cost vary based on the clinicians responsible for performing the procedure and supplies. A time-driven, activity-based, cost model with a board-certified allergist or immunologist performing outpatient PST with a one-step amoxicillin oral challenge resulted in a base case cost of $220 [37]. Personnel, supply, and space costs were $97.96, $119.08, and $2.82, respectively. However, the estimated cost decreased to $168 if a registered nurse prepared the supplies and a nurse practitioner performed the procedure. This cost is balanced with the direct cost savings after PST by using beta-lactam antibiotics instead of alternative antibiotics that may be more expensive or unnecessarily broad-spectrum [35]. Furthermore, indirect cost benefits include a lower risk of hospital-acquired infections, decreased hospital length of stay, and less emergency department and outpatient provider visits [35,39].

### 5.3. PST Coding, Billing, and Reimbursement

Outpatient PST is a reimbursable process whereas inpatient testing is part of hospitalization costs and dependent on diagnostic related grouping reimbursement. PST uses current procedural terminology (CPT) code 95018 for each prick/intradermal test, and CPT 95076 for the oral ingestion challenge that requires up to 61–120 min of observation. Code 95018 is reimbursed $18.95 on average by Centers for Medicare and Medicaid Services and would total $170.55 for four skin pricks and 5 intradermal tests (personal communication with ALK-Abello, Inc., Round Rock, TX, USA). Evaluation and Management codes L1 to L5 are also used, as appropriate, based on new patients, follow-up, and number of visits utilized. There are International Classification of Diseases, Tenth Revision codes T36.OX5A, T36.OX5D, Z88.0, and Z87.892 for adverse effect of penicillins initial encounter, subsequent encounter, allergy status, and personal history of anaphylaxis, respectively.

## 6. Penicillin Allergy Documentation

Allergy documentation in the electronic medical record (EMR) is an important component of PST. The ideal approach to allergy documentation varies depending on the health system involved. Once PST has been performed, most institutions resolve and inactivate the penicillin allergy from the EMR. It is important to input the date and results of PST into the patient’s problem list and insert a placeholder in the allergy section of the EMR with the same information in an effort to prevent relabeling of the penicillin allergy. Some institutions also have the capability to add in an electronic alert that will appear if a provider is entering a penicillin allergy in the allergy section of a patient’s chart who previously had a negative PST [40,41]. In order to further assist in the prevention of penicillin allergy relabeling, PST programs need to take a multi-faceted approach that involves communication to patient and their families, providers within and outside the healthcare network, and community pharmacies. However, this can be challenging since this process can involve several healthcare professionals and family members at various locations.

There are also several potential opportunities for allergy documentation errors to occur since multiple members of the healthcare team have access to modify these records [42]. Blumenthal and colleagues observed that allergy deletions occurred infrequently by healthcare team members and 50% of allergy alerts occurred for medications that patients tolerated previously [42]. Furthermore, the penicillin allergy may reappear with subsequent healthcare encounters if allergy record updates following PST are not documented correctly and comprehensively. After a negative PST, 49% (26/53) of patients did not have their penicillin allergy removed from the medical record [43]. An observational study in a healthcare system composed of hospital, LTCF, and primary care providers found that 36% (20/55) of patients with a negative PST had their allergies re-documented with a median follow-up period of one year. Interestingly, all 21 of the LTCF patients still had a penicillin allergy documented in the EMR [44]. Documentation errors also occur in the pediatric population. Vyles and colleagues performed a follow-up study of 100 parents and primary care providers of children with a negative PST performed in a pediatric emergency department [45]. Of 98 respondents, 52% of children still had a penicillin allergy documented in the primary care physician’s office, despite 80% (65/81) of parents reporting that they notified the primary care physician of the negative test. Thus, accurate allergy documentation following PST and communication of these results to other healthcare professionals is essential to prevent inaccurate penicillin allergy re-labeling and improve future antibiotic use.

Bourke and colleagues studied a subgroup of patients before and after the implementation and dissemination of detailed instructions to both the patient and primary care provider [46]. The printed instructions contained each antibiotic tested and clear recommendations on the specific antibiotics that should be used and avoided based on the patient’s PST results. This document improved adherence to allergy label modifications in the PST negative groups following recommendation with 68% (74/109) before and 85% (46/54) after with a median follow-up period of 15 months (*p* = 0.02). The results of PST should also be communicated to patients’ community pharmacies. If this step is omitted and a patient presents to the pharmacy with a prescription for a beta-lactam antibiotic with a penicillin allergy listed in the patient profile, a potentially unnecessary phone call will be made by the pharmacist to the provider to clarify the beta-lactam prescription or recommend an alternative antibiotic. Some PST programs also perform a post-test telephone call by a pharmacist to other healthcare professionals to reinforce the results of PST [41].

## 7. Education

### 7.1. Patient Education

While appropriate allergy documentation and communication of PST results across the continuum of care are important, patient education that clearly explains the results and ramifications of the PST procedure is also necessary to improve the accuracy and longevity of allergy documentation. If this is not done effectively, patients with a negative PST will continue to label themselves as penicillin allergic and refuse beta-lactam antibiotics. A follow-up study of 784 allergy clinic patients who had PST and a 5-day oral amoxicillin challenge were reviewed for subsequent penicillin use after PST. Of the 163 patients who did not receive a penicillin during the follow-up period, 45 (27%) refused to use a penicillin because they believed that it would not be safe or did not fully understand the PST results [47]. A similar study was performed at a tertiary academic outpatient practice over a 3-year period [48]. A total of 41% (12/29) of patients who were PST negative continued to avoid beta-lactam antibiotics. The survey reported this was related to either their personal (42%) or primary care physician’s beliefs (58%). Interestingly, six of the 12 patients would also inform a new provider that they had a penicillin allergy. PST negative patients were also asked if they were aware of the antibiotics they could be prescribed. Of the 29 patients, 18 stated all beta-lactam antibiotics, 5 stated penicillin only, and 6 did not know. This represents an excellent opportunity for pharmacists and other clinicians to provide education on beta-lactam antibiotics that patients are likely to tolerate following a negative PST. As such, it is necessary to ensure that patients are aware of and can interpret the results of a negative PST to help them serve as their own healthcare advocates [49].

Wallet cards following PST may also assist in patient education as well as disseminating PST results to other healthcare professionals [40]. These cards may also contain the generic and brand names of beta-lactam antibiotics that PST revealed the patient should tolerate. This additional information will provide clarity to other providers and pharmacies in an effort to avoid interpretation errors. A city-wide antimicrobial management program in Savannah, GA, provides information to healthcare providers on beta-lactam antibiotics (penicillins, cephalosporins, and carbapenems) that were previously tolerated by patients [40]. However, there is still room for improvement in identifying more effective educational strategies to explain the risks associated with reported penicillin allergies.

### 7.2. Clinician Education

Primary care providers offices are considered the central repository for allergy information in the EMR. The results of inpatient/outpatient PST results are commonly forwarded to primary care providers. This information is then forwarded to community pharmacies, specialists, and hospitals. Studies have indicated that despite the results of negative PST, primary care physicians do not feel comfortable prescribing beta-lactam antibiotics [50]. This may be due to a lack of knowledge on the sensitivity and specificity of PST results and the lack of drug allergy education in their training programs [51]. To assess providers knowledge of penicillin allergies and interpretation of PST results, Prematta and colleagues conducted a survey to review physician responses to a variety of allergy case scenarios [52]. The authors concluded that many physicians are unaware of the utility of a PST and this is an area where further education would be valuable. As part of the Antibiotic Prescribing and Use in Doctor’s Offices, the Centers of Disease Control and Prevention developed physician education on the evaluation and diagnosis of a penicillin allergy that highlights the negative predictive value that approaches 100% following PST with the major and minor determinant with a subsequent direct, oral challenge [10]. Furthermore, the University of South Carolina College of Pharmacy offers a 15-h penicillin allergy assessment and skin testing certificate program to train and certify clinicians [53]. This represents an excellent opportunity for clinicians to take advantage of to improve their knowledge of the issues surrounding penicillin allergies and to perform appropriate penicillin allergy assessments and PST.

## 8. Penicillin Allergy Patient Advocacy and the Role of the Pharmacist the Outpatient Setting

The outpatient setting serves as an excellent opportunity to promote patient advocacy and education of penicillin allergies. As previously described, healthcare professionals such as primary care providers, pharmacists, and nurses in the outpatient setting should support and provide penicillin allergy educational strategies for patients. Education may include, but is not limited to penicillin allergy statistics, potential unintended consequences of labeled penicillin allergies, and referral locations where a structured penicillin allergy assessment and PST may be performed. Providers and nurses within patients’ primary care providers’ offices and patients’ community pharmacists serve as an excellent mechanism to promote penicillin allergy awareness and education in the outpatient setting. Public information dissemination via different media platforms such as news and radio stations are also another strategy to reach patients in the outpatient setting in an effort to increase awareness of the concerns associated with penicillin allergies. Patients could be encouraged to be their own healthcare advocates and pursue a formal penicillin allergy assessment. This proactive approach is important for PST negative or penicillin de-labeled patients to be involved and informed of their allergy history while helping disseminate their results to healthcare professionals.

To date, pharmacist involvement in PST services has been predominately performed in the inpatient setting where the results will directly impact clinical decision making for antibiotic therapy [18]. PST is generally considered a safe service, especially when scratch testing is performed before intradermal testing and serves an excellent opportunity to potentially de-label penicillin allergies through a non-acute approach in the outpatient setting. Board-certified allergists have primarily been responsible for screening, performing, and interpreting PST for patients in the outpatient setting. However, allergists are a specialty service that are often unavailable in several healthcare systems. As such, this provides a great opportunity for pharmacists to be involved with PST in the outpatient setting. To date, the role of pharmacists in outpatient PST has been limited due to challenges including on scope of practice, billing, education, training, and personnel resources. However, pharmacists are well-positioned to be involved in PST services given their drug expertise, involvement within the healthcare team, and patient interaction opportunities. Pharmacists also practice in a wide array of outpatient practice settings including, but not limited to, community pharmacies, ambulatory care, and urgent care clinics that commonly serve diverse patient populations.

The community pharmacy setting is often recognized by patients as their first healthcare professional resource since patients can readily seek advice and counseling from a licensed pharmacist. Pharmacists in this role serve as an excellent resource to educate patients on penicillin allergies, perform structured allergy assessment interviews, and evaluate the potential opportunity for PST. Pharmacists are already counseling patients on medications, and this may be an excellent opportunity to screen patients with a labeled penicillin allergy for an appropriate allergy assessment interview. Based on the penicillin allergy assessment interview, community pharmacists can clarify incomplete or inaccurate allergy data since they have access to add, edit, and remove allergy information in a patients’ prescription record as well as the EMR in certain health systems. Pharmacists, especially those practicing in the community settings, should also take an active role in increasing referral rates to available PST outpatient clinics. One strategy to accomplish this may be to identify penicillin allergic patients from electronic prescribing records and provide them with a community resource outlining a list of available clinics that perform PST. As such, community pharmacies may be encouraged to develop a professional relationship with local allergists for potential referrals for PST.

The role of pharmacists in the community setting are also continuing to expand by administration of several immunizations and various clinical services including point-of-care testing and screening. PST may serve as a future opportunity for community pharmacists to perform, yet certain barriers currently exist that hinder this practice. As discussed previously, pharmacists’ scope of practice varies among different State Board of Pharmacies, and PST services are primarily restricted to the inpatient setting. Professional pharmacy organizations are well-positioned to advocate for pharmacists to perform PST, especially as outpatient antimicrobial stewardship continues to grow. Time and space logistics can also be challenging for community pharmacists. However, many community pharmacies already have a private immunization or consultation room available that may offer sufficient space to perform PST. While PST can be time consuming with taking 60 min or more, the majority of this time requirement is spent observing the patient for a reaction. During this time period, pharmacists can still perform their other responsibilities with periodic check-ins for observation of a reaction. Pharmacy technicians and ancillary staff can also help prepare or batch the contents necessary to perform PST so that they are readily available for the pharmacist to perform the service when needed.

While a pharmacy may view PST as a financial disincentive from the loss of the pharmacists’ time and supplies required, these costs may be further offset by decreased healthcare utilization costs and offer a potential billing opportunity for a clinical service performed by pharmacists [18,54]. Pharmacists providing service in the ambulatory care and urgent care clinic setting may also be well-positioned to perform PST with similar considerations. Although barriers to implementation of pharmacists performing PST currently exist, pharmacists and professional pharmacy organization can continue to advocate for PST due to the potential clinical and economic benefits for patients and pharmacies as part of antimicrobial stewardship endeavors in the outpatient setting [40].

## 9. Conclusions

While PST and penicillin allergy assessment has largely been performed in acute care settings where results will immediately impact antibiotic therapy decisions, there is an increasing role for implementation in the outpatient setting to improve antibiotic prescribing. Pharmacists are well-positioned to be involved in PST and penicillin allergy assessments as part of antimicrobial stewardship initiatives in the outpatient setting. However, more data and strategies to mitigate barriers to these approaches are necessary to further advance these clinical and economic benefits for patients.

## Figures and Tables

**Table 1:** Cost of specific penicillin skin testing contents.

Drug Contents of Penicillin Skin Testing	Average Wholesale Price ($)
Benzylpenicilloyl polylysine (0.25 mL) ampule (PRE-PEN)	$138.00
Penicillin G Potassium 10,000 IU/mL (5 million units)	$15.19
Amoxicillin 500 mg (250 mg/5 mL)	$0.06 per mL
Amoxicillin 500 mg capsule	$0.19–5.88
Histamine base (1 mg/mL) 5 mL	$204.40 ($2.00 per test)
